# Electronic Intervention for Patient-Managed Benzodiazepine Tapering: A Randomized Clinical Trial

**DOI:** 10.1001/jamanetworkopen.2025.51807

**Published:** 2026-01-14

**Authors:** Keith Humphreys, Hildi Hagedorn, Xiaotong Han, Lakiesha Kemp, Nichols Poitra, Michael A. Cucciare

**Affiliations:** 1 Center for Innovation to Implementation, Veterans Affairs Palo Alto Health Care System, Menlo Park, California; 2 Department of Psychiatry, Stanford University, Stanford, California; 3 Center for Care Delivery and Outcomes Research, Minneapolis Veterans Affairs Health Care System, Minneapolis, Minnesota; 4 Department of Psychiatry, University of Minnesota Medical School, Minneapolis; 5 Center for Mental Healthcare and Outcomes Research, Central Arkansas Veterans Affairs Healthcare System, North Little Rock; 6 Veterans Affairs South Central Mental Illness Research, Education and Clinical Center, Central Arkansas Veterans Healthcare System, North Little Rock; 7 Department of Psychiatry, University of Arkansas for Medical Sciences, Little Rock

## Abstract

**Importance:**

More than 30 million people in the US take prescribed benzodiazepines, which, when taken long-term, carry risks of falls, cognitive decline, and dependence. A previous trial showed that a patient-focused self-management intervention (Eliminating Medications Through Patient Ownership of End Results; EMPOWER) can reduce long-term benzodiazepine dependence use and the risks that accompany it.

**Objective:**

To replicate the finding of the EMPOWER trial after converting the intervention from printed materials to electronic format.

**Design, Setting, and Participants:**

This 2-arm, individually randomized clinical trial with a 6-month follow-up was conducted at US Veterans Health Administration primary care clinics in 2 Veterans Affairs health care systems. Participants included 161 primary care patients taking benzodiazepines for 3 or more months and having access to a smartphone, tablet, or desktop computer. Recruitment occurred from June 1, 2022, to January 31, 2024.

**Intervention:**

Participants were randomized to either the electronically delivered EMPOWER (EMPOWER-ED) protocol or asked to continue to follow clinician recommendations regarding their benzodiazepine use (treatment as usual).

**Main Outcomes and Measures:**

Preregistered primary outcomes were complete benzodiazepine cessation and at least 25% dose reduction at 6-month follow-up, assessed using pharmacy data. Secondary outcomes were self-reported anxiety symptoms, sleep quality, and overall health and quality of life. Analysis was performed on an intent-to-treat basis.

**Results:**

The 161 participants had a mean (SD) age of 61.9 (13.7) years and were mostly male (134 [83.2%]). Individuals assigned to the EMPOWER-ED group had a significantly greater likelihood of complete benzodiazepine cessation (odds ratio [OR], 5.31 [95% CI, 1.12-25.12]). There was no likelihood of at least a 25% dose reduction in the EMPOWER-ED group relative to the control group (OR, 2.51 [95% CI, 0.91-6.90]). No statistically significant difference was found between the 2 groups for the secondary outcomes.

**Conclusions and Relevance:**

This randomized clinical trial found a large effect of a low-cost, self-administered electronic intervention for reducing benzodiazepine use among long-term users. Findings from this replicated clinical trial are encouraging given the prevalence of benzodiazepine dependence and the constraints on clinician time available to address it. Dissemination of this intervention—which is in the public domain—by health care and public health systems seems warranted.

**Trial Registration:**

ClinicalTrials.gov Identifier: NCT04572750

## Key Points

**Question:**

Could the benefits of a patient-focused self-management intervention promoting benzodiazepine cessation be replicated in a second trial that converted it from paper and pencil to electronic format?

**Findings:**

In this randomized clinical trial of 161 primary care patients, the odds of benzodiazepine cessation at 6-month follow-up was 5.31 times higher among those who received the intervention than those not receiving the intervention, a significant difference, and the odds of at least a 25% reduction in benzodiazepine dose was 2.51 times higher in the intervention group, a nonsignificant difference.

**Meaning:**

This study suggests that a freely accessible, patient-directed website tool can aid benzodiazepine cessation.

## Teaser

This randomized clinical trial examines whether the benefits of a patient-focused self-management intervention promoting benzodiazepine cessation could be replicated when the intervention was converted from paper and pencil to electronic format.

## Introduction

Low-cost, electronically delivered interventions are increasingly being used to support self-managed reduction in the use of potentially addictive prescribed medications (eg, opioids)^1^ and nonprescribed drugs.^2,3,4,5,6^ One underappreciated target for such interventions is long-term benzodiazepine use, which can increase risk of cognitive decline, falls, and motor vehicle accidents.^7,8,9^ Patients can also develop dependence on benzodiazepines as well as experience risk of overdose, particularly if they also consume alcohol and/or illicit or nonillicit opioids.^10,11,12^

The Eliminating Medications Through Patient Ownership of End Results (EMPOWER) trial^13^ reported an 8-fold increase in benzodiazepine cessation among people receiving printed self-management materials and tapering strategies vs control participants. This is a large effect size for a simple intervention, but many trial findings do not replicate. EMPOWER has since been used successfully in nonrandomized quality improvement efforts,^14^ but it has never been subjected to a randomized replication trial. Furthermore, since the EMPOWER study was published, electronically delivered interventions have become more dominant given their cost and scalability advantages over printed materials.

Accordingly, the present study attempted to replicate the EMPOWER trial findings after converting the intervention to an electronic format (hereafter, *EMPOWER electronically delivered* [*EMPOWER-ED*]). Simultaneously, the intervention was tailored with content specific to US military veterans for a trial within that population, in which long-term benzodiazepine use and associated complications are prevalent.^12^ The process through which EMPOWER-ED was tailored to veterans has been described elsewhere,^15^ as has the full clinical trial protocol.^16^ In addition to attempting to replicate EMPOWER’s effect on benzodiazepine use, the EMPOWER-ED trial also evaluated potential effects on outcomes that might be affected by changes in benzodiazepine use, namely, anxiety symptoms, sleep disturbance, and overall health or quality of life.

## Methods

### Human Subjects and Preregistration

This randomized clinical trial was approved by the Veterans Affairs (VA) Central Arkansas Veterans Health Care System Research and Development Committee and Institutional Review Board (trial protocol in Supplement 1). Study staff members obtained oral consent from those expressing an interest in participating and a willingness to be followed up 6 months after randomization. Participants received $30 for completing a baseline interview and an additional $30 for completion of the 6-month follow-up interview. The study was registered at ClinicalTrials.gov (NCT04572750). The Consolidated Standards of Reporting Trials (CONSORT) reporting guideline was followed.

### Study Design and Setting

Patients at the Central Arkansas and Minneapolis Veterans Healthcare Systems with at least 1 primary care appointment in the past year were screened for eligibility. Both health care systems provide primary care at a central medical center as well as through community-based clinics throughout their catchment area.

### Participants

Potentially eligible participants who had an active benzodiazepine prescription for at least 3 months were identified using VA’s centralized health care databases. Individuals were excluded from recruitment efforts if the databases showed that they were receiving palliative care or had a current diagnosis of dementia, schizophrenia, seizure disorder, and/or spinal cord injury. Because pill splitting is often necessary when tapering is undertaken, patients taking benzodiazepines in capsule form were also excluded. Study staff members contacted and screened potentially eligible individuals for other study exclusion criteria that could not be determined through the centralized patient database, such as acute suicidality and lack of access to a smartphone or tablet. The Figure shows participants’ flow through the trial.

**Figure.**
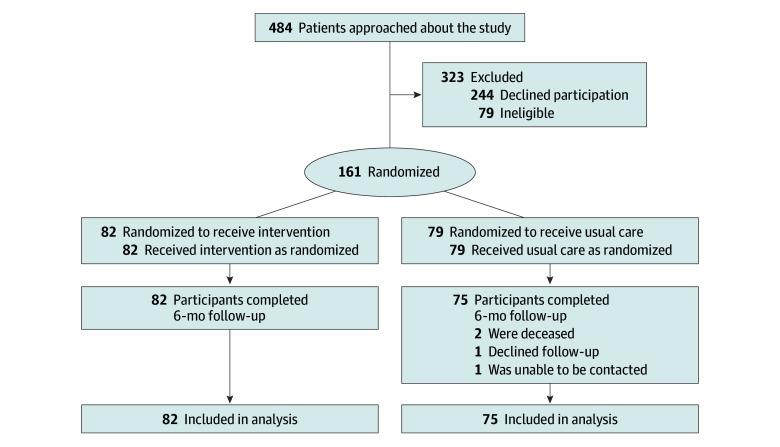
Study Flow Diagram

### Recruitment

Recruitment was conducted from June 1, 2022, to January 31, 2024. Potentially eligible participants were mailed a study invitation letter and then contacted by telephone.

### Sample Size

As described in the published protocol,^16^ based on the original EMPOWER study’s findings and a meta-analysis of clinical trials of similar interventions,^17^ power analysis indicated a need for 85 participants per group for a total sample of 170 to attain 80% power to detect a medium effect size of 0.35. Due to COVID-19–related delays in recruitment, 9 patients initially deemed eligible based on VA electronic health record data had to be excluded because they ceased benzodiazepine use before their baseline interview. Study recruitment resources thus expired with 161 participants. However, the original power analysis assumed a 20% attrition rate, whereas the actual rate was much lower (3%), so the study ultimately had more paired observations for analysis (156 vs 136) than was foreseen when the power analysis was conducted.

### Randomization and Blinding

Research staff members, guided by a biostatistician, generated and stored a randomized list of participants on an encrypted electronic file to which the principal investigators did not have access. Once they had consented, participants completed a baseline interview by telephone prior to being randomized by our biostatistician. Thus, research assistants conducting the baseline and follow-up interviews were blinded to participants’ allocation.

### Experimental Intervention

Research staff provided the EMPOWER-ED website information over the telephone, and/or by email or text to participants assigned to the experimental intervention. Participants in the experimental intervention group were allowed to access EMPOWER-ED on any platform (eg, smartphone, desktop, and tablet) that they wished. EMPOWER-ED, like the intervention on which it was based, comprises self-assessment of risks associated with long-term benzodiazepine use and information on possible benzodiazepine-related harms, including drug interactions. To support patients’ motivation and confidence in their ability to change, EMPOWER-ED offered vignettes of peers who had successfully stopped using benzodiazepines. The intervention also provided the details about other therapeutic alternatives for managing sleep difficulties and/or anxiety as well as links to mobile applications with this purpose. Participants in the experimental intervention group also received recommendations for self-tapering should they choose to reduce or abstain from benzodiazepine use and a personalized tapering schedule based on their current dose level and desired start date to initiate a taper.

### Control Group

Research staff members recommended that individuals in the control group follow any clinician recommendations regarding their benzodiazepine use but were otherwise provided no intervention. Participants in the control group were informed that if the EMPOWER-ED materials proved beneficial, they would be made available to them at the end of the study. Because the study did identify a therapeutic effect of EMPOWER-ED, all participants in the control group were emailed a link to its website once the study results were determined.

### Data Collection

All participants were interviewed over the telephone by research staff members for 30 to 45 minutes at baseline. At 6-month follow-up, 157 participants (97.5%) were relocated and reinterviewed (Figure). Data collected at the 6-month follow-up interview were supplemented with pharmacy data from the VA electronic record on benzodiazepine prescriptions received since the baseline interview.

### Outcomes

We preregistered 2 primary outcomes: cessation of benzodiazepines and a 25% or greater dose reduction of benzodiazepines at the 6-month follow-up. Cessation (yes or no) was defined as the absence of a benzodiazepine prescription renewal in the 3 months prior to the 6-month follow-up, and dose reduction (yes or no) was defined as a 25% or greater dose decrease in benzodiazepine use (including complete cessation) sustained in the 3 months prior to the 6-month follow-up. All doses of the different benzodiazepines used were converted to diazepam equivalents, and the baseline dose was defined as the mean daily dose per day in the 6 months prior to randomization.

Preregistered secondary outcomes were anxiety symptoms, sleep quality, and overall health and quality of life collected at baseline and at 6-month follow-up. Anxiety symptoms were measured using the 7-item Generalized Anxiety Disorder Scale (GAD-7).^18^ This 7-item scale asks about how often participants were experiencing symptoms (eg, feeling on edge or having trouble relaxing) in the past 2 weeks. Item severity is measured by responses ranging from 0 (not at all) to 3 (nearly every day). The 7 items are summed to obtain a total score indicating the severity of anxiety symptoms.

The Patient-Reported Outcomes Measurement System (PROMIS) was used to measure sleep disturbance.^19^ This 8-item scale assesses the degree of difficulty or trouble people have falling or staying asleep (My sleep was restless; from 1 [not at all] to 5 [very much]) and the quality of their sleep (I got enough sleep; from 1 [very much] to 5 [not at all]; My sleep quality was 1 [very good] to 5 [very poor]) over the past 7 days. PROMIS items are summed to obtain an overall raw score of sleep disturbance and converted to a T-score, with higher scores representing more sleep disturbance.

Overall health and quality of life were measured using the RAND Veterans 12-Item Health Survey (VR-12).^20^ The VR-12 comprises 12 items assessing emotional and physical well-being and functioning (In general, would you say your health is … [responses range from poor to excellent]; How much did pain interfere with your normal work? [responses range from not at all to extremely]). Two summary measures were generated describing overall physical and mental health, with higher scores representing better functioning in each domain.

### Covariates

The study measured variables with known or suspected associations with the outcomes. Specifically, data on participants’ demographic characteristics were collected by study staff members via their self-report. Demographic data collected included participants’ age, sex (female or male), and race and ethnicity (African American or Black, American Indian or Alaska Native, Native Hawaiian or Pacific Islander, White, and other [>1 race and refused to answer]). The race and ethnicity variable was regrouped into White and non-White due to White being the majority. Race and ethnicity were reported because they may influence prescription of and misuse of benzodiazepines.^21^ We also collected data on participants’ educational level (high school or less, 1-4 years of college, and ≥5 years of college); marital status (married or in a relationship, single, divorced, and widowed); housing status (yes or no); and work status (disabled, retired, unemployed or other, and working). Data were collected on participants’ alcohol use using the 3-item Alcohol Use Disorders Identification Test–Consumption screener for problem drinking (AUDIT-C; How often did you have a drink containing alcohol in the past year? [range, 0 (never) to ≥4 times per week]).^22^ Participants completed the Severity of Dependence Scale,^23^ which consists of 5 items (During the last month, did you wish you could stop? [range, 0-3, where 0 indicates never or almost never and 3 indicates always or nearly always]) measuring the severity of benzodiazepine dependence.

### Statistical Analysis

Analysis was performed on an intent-to-treat basis. We first tested study randomization by conducting bivariate analyses comparing baseline values of covariates between the 2 groups. A χ^2^ independence test was used for testing categorical variables, 2-sample independence tests were used for normally distributed continuous variables, and Wilcoxon rank sum tests were used for nonnormally distributed continuous variables. Participants’ AUDIT-C scores were the only variable that emerged as significantly different between groups. However, participants’ AUDIT-C scores did not influence model results testing outcomes, so they were not included in the final models.

Descriptive statistics for the primary and secondary outcomes and for covariates were calculated for both study groups. Logistic regression models were used to examine the effect of the EMPOWER-ED intervention on the primary outcomes of benzodiazepine cessation and dose reduction (≥25% dose reduction) at the 6-month follow-up. The binary study group variable (EMPOWER-ED vs control) was included in the model as the independent variable. For adjustment, the baseline benzodiazepine dose per day was also included in models testing outcomes. For each model, odds ratios (ORs) were calculated along with the corresponding 95% CI.

A total of 11% of participants (18 of 161) had missing data for the secondary outcomes (anxiety, sleep, and quality of life) at the baseline interview. Therefore, to examine the associations between the study group variable and the secondary outcomes, missing data at baseline were imputed using multiple imputation methods under the assumption that data are missing randomly. Fifteen imputed datasets were created. The analysis was conducted within each of the 15 imputed datasets, and the final results (eg, parameter estimates) were obtained using the mean values from the 15 imputed datasets. General linear mixed models were used for normally distributed outcomes (quality-of-life indicators and sleep quality), and the generalized linear mixed model was used for examining anxiety symptoms, with multinomial distribution and cumulative logit specified. The models included study group as the independent variable, time representing baseline, the 6-month follow-up, and the time-by-study group interaction. A 2-sided significance level of *P* < .05 was used for hypothesis testing and 95% CI calculation. SAS, version 9.4 (SAS Institute Inc) was used for all analyses.

## Results

### Descriptive Statistics and Bivariate Analysis

Table 1 shows the descriptive statistics for the demographic characteristics for each study group. The mean (SD) age of participants was 61.9 (13.7) years, and the total cohort included 134 men (83.2%) and 27 women (16.8%) and 11 African American or Black participants (6.8%), 1 American Indian or Alaska Native participant (0.6%), 1 Native Hawaiian or Pacific Islander participant (0.6%), 143 White participants (88.8%), and 5 participants who were of multiple races or ethnicities or refused to answer (3.1%). Most participants had some college education, were married or in a relationship, had housing, and were retired or disabled.

**Table 1.** Descriptive Statistics for Demographic Characteristics by Each Study Group

Characteristic	Participants, No. (%)		
	Control (n = 79)	EMPOWER-ED (n = 82)	Total (N = 161)
Site			
Little Rock	50 (63.3)	53 (64.6)	103 (64.0)
Minneapolis	29 (36.7)	29 (35.4)	58 (36.0)
Age, mean (SD), y	61.5 (13.9)	62.3 (13.6)	61.9 (13.7)
Sex			
Female	16 (20.3)	11 (13.4)	27 (16.8)
Male	63 (79.8)	71 (86.6)	134 (83.2)
Race and ethnicity			
African American or Black	6 (7.6)	5 (6.1)	11 (6.8)
American Indian or Alaska Native	1 (1.3)	0	1 (0.6)
Native Hawaiian or Pacific Islander	0	1 (1.2)	1 (0.6)
White	68 (86.1)	75 (91.5)	143 (88.8)
Other^a^	4 (5.1)	1 (1.2)	5 (3.1)
Educational level			
High school or less	11 (13.9)	23 (28.1)	34 (21.1)
College 1-4 y	48 (60.8)	40 (48.8)	88 (54.7)
College ≥5 y	20 (25.3)	19 (23.2)	39 (24.2)
Marital status			
Married or in a relationship	48 (60.8)	44 (53.7)	92 (57.1)
Single	9 (11.4)	14 (17.1)	23 (14.3)
Divorced	19 (24.1)	18 (22.0)	37 (23.0)
Widowed	3 (3.8)	6 (7.3)	9 (5.6)
Housing			
Yes	78 (98.7)	81 (98.8)	159 (98.8)
Work status			
Disabled	28 (34.9)	25 (30.5)	53 (32.6)
Retired	30 (38.0)	39 (48.0)	69 (43.1)
Unemployed or other	4 (5.2)	2 (2.4)	6 (3.8)
Working	17 (22.0)	16 (19.1)	33 (20.5)
Baseline AUDIT-C score, mean (SD)	0.8 (1.6)	1.6 (2.6)	1.2 (2.2)
Baseline dependence severity score, mean (SD)	2.7 (2.5)	3.4 (2.8)	3.1 (2.7)

Abbreviations: AUDIT-C, Alcohol Use Disorders Identification Test–Consumption items; EMPOWER-ED, Eliminating Medications Through Patient Ownership of End Results–electronically delivered.

^a^ Includes participants reporting multiple races and those declining to answer.

Table 2 shows primary and secondary outcomes along with the baseline clinical characteristics. Bivariate analysis was performed between study groups. Participants in the EMPOWER-ED group reported a baseline mean (SD) daily benzodiazepine dose (in diazepam equivalents) of 22.7 (20.2) mg, whereas participants in the control group reported a mean (SD) daily dose of 22.0 (18.2) mg. For the primary outcomes, the participants in the EMPOWER-ED group reported a significantly higher proportion of benzodiazepine cessation than the control group (10 of 82 [12.2%] vs 2 of 79 [2.5]; unadjusted OR, 5.35 [95% CI, 1.13-25.24]) (Table 2). There was no likelihood for more participants in the EMPOWER-ED group to report benzodiazepine dose reduction of 25% or greater than in the control group (14 of 82 [17.1%] vs 6 of 79 [7.6]; unadjusted OR, 2.50 [95% CI, 0.91-6.90]).

**Table 2.** Descriptive Statistics for Primary and Secondary Outcomes by of Each Study Group

Variable	Control (n = 79 [49.1%])	EMPOWER-ED (n = 82 [50.1%])	Total (N = 161 [100%])	*P* value
**Primary outcomes (at 6-mo follow-up)**				
Benzodiazepine cessation, No. (%)^a^	2 (2.5)	10 (12.2)	12 (7.5)	.02
Benzodiazepine dose reduction ≥25%, No. (%)^b^	6 (7.6)	14 (17.1)	20 (12.4)	.07
**Secondary outcomes**				
VR-12 Physical health score, mean (SD)				
Baseline	38.0 (13.4)	38.1 (11.3)	38.0 (12.3)	.91
Follow-up	37.3 (13.0)	37.5 (10.8)	37.4 (11.9)	.92
VR-12 Mental health score, mean (SD)				
Baseline	40.2 (12.1)	40.2 (13.5)	40.2 (12.8)	.89
Follow-up	41.3 (10.2)	40.7 (11.6)	41.0 (11.0)	.73
Anxiety (GAD-7 score), median (range)				
Baseline	9 (0-21)	10 (0-21)	9 (0-21)	.34
Follow-up	6.5 (0-21)	7.5 (0-21)	7 (0-21)	.37
Sleep (PROMIS scale score), mean (SD)				
Baseline	24.5 (9.7)	23.8 (8.6)	24.2 (9.1)	.67
Follow-up	23.6 (7.9)	23.8 (8.5)	23.7 (8.2)	.90
**Baseline clinical characteristics**				
Baseline AUDIT-C score, mean (SD)	0.8 (1.6)	1.6 (2.6)	1.2 (2.2)	
Baseline dependence severity score, mean (SD)	2.7 (2.5)	3.4 (2.8)	3.1 (2.7)	

Abbreviations: AUDIT-C, Alcohol Use Disorders Identification Test–Consumption items; EMPOWER-ED, Eliminating Medications Through Patient Ownership of End Results–electronically delivered; GAD-7, 7-item Generalized Anxiety Disorder; PROMIS, Patient-Reported Outcomes Measurement Information System (8 items); VR-12, Veterans RAND 12-Item Health Survey.

^a^ Unadjusted odds ratio, 5.35 (95% CI, 1.13-25.24).

^b^ Unadjusted odds ratio, 2.50 (95% CI, 0.91-6.90).

For the secondary outcomes, participants in the control group reported a mean (SD) VR-12 physical health component score of 38.0 (13.4) at baseline and 37.3 (13.0) at follow-up and a mean (SD) VR-12 mental health component score of 40.2 (12.1) at baseline and 41.3 (10.2) at follow-up (Table 2). Participants in the EMPOWER-ED group reported a mean (SD) VR-12 physical health component score of 38.1 (11.3) at baseline and 37.5 (10.8) at follow-up and a mean (SD) mental health component score of 40.2 (13.6) at baseline and 40.7 (11.6) at follow-up. No statistical significance was found between the study groups for these variables at baseline or follow-up. VR-12 component scores lower than 50 indicate a below-average health-related quality of life, relative to the general population, in each domain.^20^

EMPOWER-ED participants reported a baseline GAD-7 median score of 10 (range, 0-21), whereas those in the control group reported a baseline median score of 9 (range, 0-21) (Table 2). At follow-up, participants in the EMPOWER-ED group reported a GAD-7 median score of 7.5 (range, 0-21), and those in the control group reported a GAD-7 median score of 6.5 (range, 0-21). GAD-7 total scores of 5, 10, and 15 are routinely used as cutoff scores to indicate mild, moderate, and severe anxiety, respectively.^24^ As a measure of sleep disturbance, participants in the EMPOWER-ED group reported a mean (SD) raw PROMIS score of 23.8 (8.6) at baseline and 23.8 (8.5) at follow-up, and those in the control group reported a mean (SD) raw PROMIS score of 24.5 (9.7) at baseline and 23.6 (7.9) at follow-up. Raw scores on the PROMIS are converted to T-scores. For example, a raw PROMIS score of 25 equals a T-score of 55.3, indicating more sleep disturbance than an average person in the general population.^19^ In terms of baseline clinical characteristics, AUDIT-C scores differed by group, with participants in the EMPOWER-ED group reporting higher scores than those in the control group.

### Models for Primary and Secondary Outcomes

The logistic regression models for the primary outcomes at the 6-month follow-up adjusting for baseline mean daily dose per day showed that participants in the EMPOWER-ED group were significantly more likely than controls to report benzodiazepine cessation at the 6-month follow-up (OR, 5.31 [95% CI, 1.12-25.12]) (Table 3). Participants in the EMPOWER-ED group, relative to controls, showed no likelihood of reporting a higher rate of a 25% or greater dose reduction in their benzodiazepine use at the 6-month follow-up (OR, 2.51 [95% CI, 0.91-6.90]). The general and generalized linear mixed models for the secondary outcomes (anxiety symptoms, sleep disturbance, and quality-of-life indicators) showed that the interaction between the study group and time was not statistically significant for any of the secondary outcomes (Table 4).

**Table 3.** Logistic Regression Models for the Primary Outcomes at 6-Month Follow-Up^a^

Variable	Odds ratio (95% CI)	
	Benzodiazepine cessation	Benzodiazepine dose reduction ≥25%
Baseline benzodiazepine daily dosage per day	1.01 (0.99-1.04)	0.99 (0.98-1.02)
EMPOWER-ED group (reference = control group)	5.31 (1.12-25.12)	2.51 (0.91-6.90)

Abbreviation: EMPOWER-ED, Eliminating Medications Through Patient Ownership of End Results–electronically delivered.

^a^ No other variables were included in the models.

**Table 4.** Generalized Linear Mixed Models for the Secondary Outcomes^a^

Variable	Parameter estimates (95% CI)			
	Physical health	Mental health	Anxiety	Sleep
Group (reference = control group)	0.03 (−3.73 to 3.78)	−0.02 (−3.72 to 3.67)	0.49 (−0.55 to 1.53)	−0.69 (−3.40 to 2.01)
Time (reference = baseline)	−1.08 (−3.24 to 1.08)	0.82 (−1.59 to 3.23)	−0.80 (−1.41 to −0.19)	−0.36 (−1.90 to 1.18)
Group × time (reference = control group, baseline)	0.30 (−2.70 to 3.30)	−0.39 (−3.74 to 2.95)	−0.07 (−0.91 to 0.77)	0.33 (−1.80 to 2.45)

^a^ No other variables were included in the models.

## Discussion

The number of individuals who experience harm from prescribed and nonprescribed addictive substances markedly exceeds the ability of the health care system to provide professional care. Such individuals can take an active role in their own health, as evidenced by the accumulating data on the benefit of mutual help programs for substance use disorders^25^ and for educational materials and technology-delivered programs (websites and mobile apps) that help people self-manage their use.^17^ To our knowledge, EMPOWER is the only such intervention targeted specifically to long-term benzodiazepine users, and the replication of its benefits as well as it being technically upgraded to an easy-to-use website should increase willingness among health care policy leaders to disseminate it.

The effect size for benzodiazepine cessation was, as in the original EMPOWER trial, large, reflecting a more than 5-fold increase in cessation. This outcome was not guaranteed, given that the original EMPOWER trial included an in-person consultation visit and also that the VA has been lessening benzodiazepine prescribing in recent years, which might have left a population of long-term users who had difficulty reducing their use. Confidence that the intervention was beneficial is strengthened by the extremely high follow-up rate (97.5%) in this study, which rules out the possibility that an attrition by functioning interaction artifact could have produced the results. That the finding was not only replicated but was also of meaningful clinical size is thus highly encouraging.

A potential risk of benzodiazepine tapering is the rebound of symptoms of anxiety or sleeplessness. There was no evidence in our study that this occurred. At the same time, EMPOWER-ED did not improve these outcomes either.

### Limitations

This trial has some limitations. A focus group process was used to tailor EMPOWER-ED to veterans. Similar tailoring will be needed if the website is rolled out in health care systems serving different populations. Furthermore, because the prescribing data came from the VA, the study could not detect if participants switched their prescriptions from the VA to a non-VA clinician, producing an illusion of cessation (although this seems unlikely given the financial incentives at play). Also, other benzodiazepine use (eg, through diversion) was not captured, perhaps affecting the accuracy of some participants’ medication use.

## Conclusions

In this randomized clinical trial, we found a large effect of a low-cost, self-administered intervention for reducing benzodiazepine use among long-term users. Benzodiazepine-related risks remain prevalent and poorly addressed in the health care system. This is the second randomized clinical trial (after the original EMPOWER trial^13^) to indicate that progress can be made on this public health problem without burdensome costs. Rolling out some version of EMPOWER is thus an attractive option for health care systems and public health departments more generally.

## Notes

Supplement 1.
Trial Protocol and Statistical Analysis Plan

Supplement 2.
Data Sharing Statement
