# Supplementary material for: Electronic Intervention for Patient-Managed Benzodiazepine Tapering: A Randomized Clinical Trial
Source: JAMA Netw Open. 2026 Jan 14;9(1):e2551807. doi: 10.1001/jamanetworkopen.2025.51807 (PMC12805446; doi:10.1001/jamanetworkopen.2025.51807)
Supplement: Supplement 1. — Trial Protocol and Statistical Analysis Plan [file jamanetwopen-e2551807-s001.pdf]

Protocol Number and Title: IRBNet #1629044, Promoting Benzodiazepine Cessation via Electronically Delivered Patient Self-Management Intervention

(EMPOWER for Veterans; **Aim 2**)

Funding Agency: VA Health Services Research and Development, IIR 18-026

Investigator/Study Chair: Michael A. Cucciare, PhD

Version and Date: v6. June 7, 2023

Abstract

Background and Significance

Long-term dependency of benzodiazepine carries significant health risks among users, giving cause for alarm among populations. Although benzodiazepines can be effective in short-term use, when taken for extended periods, they create greater risks of cognitive decline and other brain damage, falls and other accidents, benzodiazepine dependence, and opioid-benzodiazepine overdose. This has become a major concern within VA, where over 350,000 Veterans are prescribed benzodiazepines annually, and two-thirds of that population takes them long-term (i.e., 3 months or more) (VA Pharmacy Benefits Management Academic Detailing Service, 2017) [5]. However, these risks might be reduced with low-cost patient self-management interventions. A paper-and-pencil version of one such intervention (Eliminating Medications Through Patient Ownership of End Results; EMPOWER) proved effective in a Canadian clinical trial with older adults (Tannenbaum et al., 2014) [15]. Ideally, digitizing such an intervention for electronic delivery and tailoring it to different populations to include Veterans could expand its reach. Accordingly, this protocol describes Aim 2 of three aims of this research study. Aim 2 specifically addresses research for a randomized controlled trial to test the effectiveness of an electronically delivered direct-to-patient intervention for promoting benzodiazepine cessation tailored to U.S. military veterans.

Specific Aims

Aim 1 has been completed. A promising non-VA benzodiazepine cessation intervention (EMPOWER) has been converted from paper & pencil to an electronic format tailored to Veterans. Aim 2, conduct **a randomized clinical trial of the effectiveness of the tailored, electronic intervention (EMPOWER for Veterans) on VA primary care patients' benzodiazepine cessation/reduction and functional outcomes is being implemented.**   A third aim will seek to conduct a budget impact analysis to estimate the costs of implementing EMPOWER for Veterans throughout VA. The Palo Alto, CA site will conduct Aim 3 and thus seek approval for related study activities at their site. **This protocol seeks approval for Aim 2 of this research study.**

Innovation

Converting the effective use of the paper-and-pencil version of EMPOWER to an electronic version that works on smart phones, tablets, and/or desktop computers, the proposed project intends to tailor the electronic version (EMPOWER for Veterans) to the needs and preferences of the Veteran population; in effect reducing risks among this population of benzodiazepine users.

Methodology

When EMPOWER for Veterans is fully designed, its effectiveness will be evaluated in a two-arm individually randomized controlled trial with 170 Veterans at the Central Arkansas Veterans Healthcare system and the Minneapolis VA Health Care System, and affiliated sites. Veterans who have been taking benzodiazepines for at least 3 months and have access to a smartphone or desktop/laptop computer will be invited to participate. The primary hypothesis of the study is that Veterans receiving EMPOWER for Veterans will be significantly more likely to cease benzodiazepine use entirely and reduce their dose by at least 25% at 6-month follow-up than the control group. The secondary hypothesis is that Veterans receiving EMPOWER for Veterans will also experience fewer anxiety symptoms, better sleep quality, and overall health/quality of

life at 6-month follow-up. A supplemental analysis of benzodiazepine use only will be conducted at 12 months, using VA databases to evaluate whether changes identified persist over time.

Impact

Considering that electronically delivered interventions are inexpensive to disseminate once they have been developed, this project has an excellent chance to promote health care value by creating an easily scaled-up, method of reducing the prevalence of the widespread risk of long-term benzodiazepine use to improve Veterans' health.

List of Abbreviations

CAVHS: Central Arkansas Veterans Healthcare System

EMPOWER: Eliminating Medications Through Patient Ownership of End Result (Paper Version)

EMPOWER for Veterans: Eliminating Medications Through Patient Ownership of End Results for Veterans (Electronic Version)

GAD-7: Generalized Anxiety Disorder Scale

PCP: Primary Care Provider

PHI: Protected Health Information

RCT: Randomized Control Trial

TLFB: Timeline Followback

VA PBM: VA Pharmacy Benefits Management

VHA: Veterans Health Administration

Table of Contents

Protocol Title: 1
1.0 Study Personnel 6
2.0 Introduction 7
3.0 Objectives 8
4.0 Resources and Personnel 8
5.0 Study Procedures 10
5.1 Study Design 10
5.2 Recruitment Methods 13
5.3 Informed Consent Procedures 13
5.4 Inclusion/Exclusion Criteria 15
5.5 Study Evaluations 15
5.6 Data Analysis 16
5.7 Withdrawal of Subjects 17
6.0 Reporting 17
7.0 Privacy and Confidentiality 18
8.0 Communication Plan 18
9.0 References 18

1.0 Study Personnel

Principal Investigators:

Michael A. Cucciare, PhD
Associate Director for Research Training, South Central Mental Illness Research, Education and Clinical Center
Core Investigator, HSR&D COIN, Center for Mental Healthcare and Outcomes Research
Building 11 16/MIR
2200 Fort Roots Drive
North Little Rock, AR 72114
501-257-1068
Michael.Cucciare@va.gov

Keith Humphreys, PhD
Center for Innovation to Implementation
VA Palo Alto Healthcare System
795 Willow Road
Menlo Park, CA 94025
Keith.Humphreys@va.gov

Co-Investigators:

Mark Bounthavong, Pharm. D., PhD
Pharmacist, Health Economist, Data Program Manager
Center for Innovation to Implementation
Palo Alto VA Health Care System
795 Willow Road
Menlo Park, CA 94025
Mark.bounthavong@va.gov

Hildi Hagedorn, PhD
Core Investigator, Director of the Implementation Core
Center for Care Delivery and Outcomes Research
Minneapolis Veterans Affairs Health Care System
One Veterans Drive
Minneapolis, MN 55417
Hildi.Hagedorn@va.gov

2.0 Introduction

Long-term dependence on prescribed benzodiazepines is a public health challenge which has been the subject of significant concern in multiple countries, including Israel, the United Kingdom, and the United States (Bachhuber et al., 2016; Lembke et al., 2018) [1-2]. Although useful as short-term medications for some patients (e.g., those experiencing transitory sleep difficulties), benzodiazepines convey significant risk, particularly when taken long-term. The range of adverse outcomes includes cognitive decline, falls, motor vehicle accidents, benzodiazepine dependence, and opioid-benzodiazepine overdose (Park et al., 2020; Sun et al., 2017; VA Pharmacy Benefits Management Academic Detailing Service, 2021) [3-5]. Further, although in the short-term benzodiazepines can relieve their most common indications – anxiety and sleep disturbance – in the long-term they often exacerbate them (Lader, 2011) [6]. Therefore, effective strategies are needed to help people discontinue long-term benzodiazepine use.

Although therapist-delivered cognitive-behavioral psychological interventions (e.g., for insomnia and anxiety) can reduce benzodiazepine use (Gould et al., 2014) [7], limits on patient willingness and health care system resources make providing individual psychotherapy to even a plurality of long-term benzodiazepine users very challenging. A less costly, more scalable, approach to reducing benzodiazepine use is to use the Internet to directly provide patients with structured materials that impart risk information and encourage them to reduce or cease consumption, either on their own or in consultation with their prescribing physician. As such interventions that do not require patients to travel and that can be accessed at any time and from virtually any place could be attractive to individuals who might not access clinic-based psychotherapy.

Interventions that operate on smartphones and desktop computers are increasingly being studied as a low-cost, highly accessible method for changing substance use behavior (Garnett et al., 2017; Cheung et al., 2020) [8,9]. Although this intervention delivery method has not to our knowledge been evaluated with benzodiazepine cessation or reduction, it has shown success among hazardous drinkers. For example, Cunningham and colleagues (Cunningham, Humphreys et al, 2009; Cunningham, Neighbors, Wild & Humphreys, 2012) [10,11] have demonstrated that heavy drinkers reduce alcohol consumption in response to short, self-administered assessments of alcohol consumption coupled with personalized feedback on risks and on variance from normative population consumption. Further, electronically delivered interventions are inexpensive to disseminate and increasingly preferred by patients (Erbes et al., 2014; Klee et al., 2016) [12,13], increasing their likelihood of broad adoption in healthcare systems (Damschroder & Hagedorn, 2011) [14].

A promising model for such an intervention comes from a Canadian clinical trial of self-management materials that provided information about the risks of long-term benzodiazepine use and suggested strategies for tapering down. The Eliminating Medications Through Patient Ownership of End Results (EMPOWER) study demonstrated that older long-term benzodiazepine users who were mailed paper and pencil materials were over five times more likely to discontinue use of benzodiazepines than were controls (Tannenbaum et al., 2014) [15]. If EMPOWER works in electronic form, it would greatly expand its potential reach.

One national health care system in which such an intervention could be particularly valuable is the US Veterans Health Administration (VHA). The VHA is a government-financed health care system which offers comprehensive care to over nine million individuals with prior service in the U.S. military. In the VHA, 355,298 Veterans were prescribed benzodiazepines in the fiscal year 2016, almost two-thirds (63.6%) of whom took them for three months or more (VA Pharmacy Benefits Management, 2017) [16]. The VHA population includes many elderly individuals and many who take prescribed opioids, further increasing the risk of long-term benzodiazepine use.

To tailor EMPOWER to the veteran population, our research team worked with VHA patients, physicians, and administrators; we then worked with software developers to convert the intervention to a computer-delivered format we call EMPOWER for Veterans (Electronic Version). This protocol describes how EMPOWER for Veterans will be tested in a randomized clinical trial evaluating whether it is effective with a markedly different population and format than in the initial Canadian study. The primary hypotheses of the RCT are that long-term users of benzodiazepines who are assigned to receive EMPOWER for Veterans will be significantly more likely than controls to cease taking benzodiazepines or reduce their benzodiazepine dose by at least 25% at six-month follow-up. The secondary hypotheses are that relative to controls, participants receiving EMPOWER for Veterans will improve more on anxiety symptoms, sleep quality, and overall health/quality of life at six-month follow-up. As functional outcomes were not measured in the original EMPOWER study, this is a unique contribution of the present trial. In addition to being important in themselves, these three secondary outcomes may moderate or mediate participants' response to EMPOWER for Veterans. For example, if secondary outcomes improve upon benzodiazepine tapering, participants may be more likely to continue to full cessation. In contrast, if functional outcomes deteriorate, patients and their physicians may be inclined to abandon EMPOWER for Veterans; if this were a common result it would likely reduce the adoption of EMPOWER for Veterans within VHA. Also, part of our secondary analysis will be to explore whether any effects of EMPOWER for Veterans observed on our primary outcomes at six-month follow-up are maintained at 12-month follow-up.

3.0 Objectives

The overall goal of this project is to create an electronic version of EMPOWER tailored to Veterans receiving VHA primary care. Specifically, this study maintains three aims. This protocol is specific to Aim 2, which is described in detail as the following:

Aim 2: Conduct a randomized clinical trial to test the effectiveness of the tailored, electronic intervention (EMPOWER for Veterans) on VA primary care patients' benzodiazepine cessation/reduction and functional outcomes at follow-up.
The primary hypotheses of Aim 2 are that in a clinical trial of 170 Veterans who are long-term users of benzodiazepines, those receiving the EMPOWER for Veterans intervention will be significantly more likely at 6-month follow-up to (a) cease taking benzodiazepines entirely, and (b) reduce their benzodiazepine dose by 25% or more. A secondary hypothesis of Aim 2 is that, relative to controls, Veterans receiving EMPOWER for Veterans will improve more on three functional outcomes: anxiety, sleep quality, and overall health/quality of life.

This protocol only describes Aim 2 research activities, which will occur at the Central Arkansas Veterans Health Care System; and as a result, IRB approval is being requested to conduct Aim 2 only.

We plan to request approval for Aim 3 at the Palo Alto Veterans Healthcare System, since the work for that aim will be conducted at that site.

4.0 Resources and Personnel

Michael A. Cucciare, PhD, Principal Investigator. Drs. Cucciare and Humphreys (Co-PI, located at Palo Alto) will share responsibility for the overall study. Dr. Cucciare will be responsible for supervising the Little Rock team. He will co-lead efforts to recruit participants and work closely with the Little Rock computer programmer to identify eligible participants for Aim 2, coordinate recruitment efforts, baseline, and follow-up interviews, and serve as a liaison to the primary care clinics at the Little Rock and Minneapolis VA. He will work with Drs.

Bounthavong and Humphreys to co-lead manuscript development, data analysis, and communicate findings to VA Central Office partners and the field.

Richard Owen, MD, Study Physician. Dr. Owen will be available to address any medical concerns that study participants may have over the course of the study. He will be available to contact any participant that notifies the study team of a physical or medical concerns.

Kathy Marchant, RN, Study Nurse, and Project Coordinator. Ms. Marchant will organize all aspects of the study, including assisting Drs. Cucciare and Humphreys in setting up study procedures; conducting baseline and follow-up interviews; coordinating study activities with the RA and maintaining an Access database to facilitate computer assisted telephone interviews. She will assist with overall project management activities and coordination of recruitment efforts. Ms. Marchant is also a registered nurse and is familiar with primary care settings. She will assist with participant call triage in collaboration and under the supervision of Dr. Richard Owen. She will work closely with Dr. Cucciare to communicate study findings to the Little Rock and Minneapolis VA primary care teams.

Lakiesha Kemp, MA, Research Assistant. Ms. Kemp will assist in operationalizing the study. She will obtain consent, manage the regulatory requirements for IRB and R&D, and work with the programmer in generating lists of potentially eligible Veterans. She will conduct baseline assessments, follow-up interviews and manage participant reimbursement.

Nick Poitra, Research Assistant. Mr. Poitra will assist with recruitment, conduct baseline and follow-up assessments and participant reimbursement.

Penny White, BS, Research Assistant. Ms. White will assist with recruitment, conduct baseline and follow-up assessments and participant reimbursement.

James (Silas) Williams, MS, Programmer. Mr. Williams will serve as the programmer for this study. He will obtain all necessary approvals to obtain study data, assist the study team members in obtaining DART approval and generate lists of potential eligible Veterans from the Corporate Data Warehouse. He will clean the data and provide to the study team. He will work with the study team, VA OIT and the contractor to ensure that the EMPOWER for Veterans is maintained on a VA server. He will also help the team troubleshoot “bugs” and other problems with the program as they arise.

Xiaotong Han, MS, Data Analyst. Ms. Han has worked as a data analyst for the HSR&D COIN in North Little Rock and Division of Health Services Research at UAMS for over 15 years. She will attend regularly scheduled team meetings to review the instruments, formalize the data analytic plan, and create study databases. She will advise the team on data entry for conducting longitudinal analysis, statistical procedures appropriate to answer the study research questions, and lead all data analytic activities.

Contractor, Blue Ridge Technologies will be assisting with the creation of EMPOWER for Veterans. Blue Ridge Technologies is known for providing information technology and consulting services to private enterprises as well as federal and state governments. Blue Ridge maintains a reputation for understanding the budgeting challenges that government often faces in trying to meet the demands of high-stake issues. As a result, this company provides quality technological assistance and solutions that render cost savings, risk reductions, and timely performance. Its core competencies include data management, big data analytics and visualization, management consulting, software and systems engineering, agile program and

project management, staffing services, and QA Center of Excellence. Blue Ridge's team will work closely with Dr. Cucciare to ensure that EMPOWER for Veterans is developed in conjunction with VA IT requirements and is compatible with VA servers.

5.0 Study Procedures

Refer to section 5.1-5.7 for a detail of study procedures.

5.1 Study Design

Study Design and Setting

This study will be a randomized controlled trial (N=170) conducted at the Central Arkansas Veterans Healthcare System (CAVHS) and the Minneapolis VA Health Care System to include their affiliated sites. Patients with at least one primary care appointment in the last year will be screened for eligibility.

Participants

We will generate a list of potentially eligible participants using VHA's centralized patient database (CDW) for both CAVHS and Minneapolis VA Health Care System.
Inclusion criteria include 1) having a designated primary care provider at CAVHS, Minneapolis VA and their affiliated sites, 2) taking prescribed benzodiazepines for three months or longer (as measured in the Electronic Health Record [EHR]), 3) taking their medication in tablet (as opposed to capsule) form, 4) internet access and a desktop computer, tablet, or smartphone.
Exclusion criteria include 1) palliative care patient, 2) current diagnosis of dementia, schizophrenia, seizure disorder and/or spinal cord injury, and 3) visual impairment (such that they are unable to read on a webpage.) Study staff will contact and screen potential participants for other exclusion criteria that cannot be determined through the centralized patient database (i.e., internet access, lack of access to a smartphone or tablet, visual impairment).

Although there is an indicator in the CDW to determine if medication is in tablet or capsule form; study staff will assess medication form during the screening process to ensure we do not inadvertently include someone taking capsule. Additionally, there is an indicator in the CDW to assess visual impairment via diagnostic codes (e.g., H53 visual disturbance, H54 blindness and low vision); however, study staff will assess via self-report during the screening process. These inclusion criteria are based on both the original EMPOWER study and feedback from stakeholders (Veterans, VA operational partners) in Aim 1 and from the CAVHS IRB review committee.

To be eligible, participants will be taking any benzodiazepine medications. Based on feedback from our operational partners, their data indicates that ~99% of Veterans are taking clonazepam, lorazepam, alprazolam, diazepam, or temazepam. Therefore, we expect that most, if not all, Veterans enrolled will be taking one of these medications. Data collected from the CDW, medication type and dose, will be coded as a crosswalk and maintained in an unidentifiable (with only study ID's) database. All inclusion and exclusion criteria will be coded as a cross walk and maintained an unidentifiable (with only study ID's) database.

Recruitment

Individuals meeting study eligibility criteria will be mailed an opt-out letter with information about the study, an invitation to participate, an option to 'opt-out' of study participation and study staff contact information. The recruitment mailing will also include a copy of the study informed

consent and HIPAA authorization forms, and a pre-addressed postage-paid envelope. Study staff will contact the individual 10-14 days after mailing the invitation letter unless the potential participants return the opt-out card. We will make up to 6 phone calls to attempt to contact the potential participant. If we do not reach them after 6 calls, they will be placed on our “do not contact” list. If a Veteran chooses to contact study team via SMS (text message), study staff will respond to the text message with request to schedule time to talk with the Veteran via VA cell phone. Study staff will inform the Veteran that SMS messaging is not encrypted and suggest that the Veteran delete sent and received text messages after receiving.

Prior to conducting informed consent, the study team will ask screening questions to confirm initial eligibility and confirm with the Veteran that they have a paper copy of the informed consent and HIPAA authorization forms in hand and agree to preferred method of obtaining ICF and HIPAA documents prior to initiation of the informed consent process.

The study team may re-share the ICF and HIPAA authorization via mail, fax, or via VA Outlook email using Azure Rights management Services (RMS) when the Veteran has the ability to print out hard copies to use in the informed consent process.

Study staff will send an unencrypted email with instructions from the User Guide for External Azure RMS Recipients first to confirm the Veteran’s email address and help recipient to open the email message. Study team will then send a second email with the informed consent and HIPAA authorization documents attached, which will include instructions on how to securely transmit pictures or scanned images using RMS.

Once the potential participant has available hard copy or DocuSign informed consent and HIPAA authorization documents, the study staff will review the study purposes, procedures, risk, and benefits and allow time for questions. Study staff will inform Veterans that if they are assigned to the EMPOWER condition, they may voluntarily choose to taper their benzodiazepines; however, this is not required to participate in the study. This is also stated on the EMPOWER website.

Participate Compensation:
Veterans will be compensated for their time, checks for $30 will be mailed to them 4-6 weeks after completing the baseline and 6-month assessments. Participants who complete both the baseline and 6-month assessments will receive a total of $60.

Sample Size
The original EMPOWER study reported a more than five-fold difference in benzodiazepine cessation and reduction rates with an OR greater than 8, which converts to a Cohen’s d greater than 0.8 Tannenbaum et al., 2014) [15]. However, we powered the present study using a more conservative estimate of the intervention effect based on a recent meta-analysis of 17 clinical trials (Boumparis et al., 2017) [17] showing that computer-delivered interventions demonstrate an effect size on drug use only somewhat lower than medium (0.36).

To attain 80% power with .05 type I error rate to detect a medium effect size of 0.35 with intra-class coefficient of .008 (based on the original EMPOWER study; Tannenbaum et al., 2014) [15] for the primary outcomes of benzodiazepine cessation/reduction, 66 patients per group will be needed. Assuming an 80% six-month follow-up rate, this will require enrollment at baseline of

170 participants (i.e., 85 per group). All data will be analyzed by the study biostatistician (Ms. Han).

Randomization

Upon receiving documentation of informed consent and HIPAA authorization, research staff will randomize participants to the EMPOWER for Veterans tool or to treatment as usual. Simple randomization of participants will be conducted using the website Randomizer.org. Research assistants, guided by a biostatistician, will generate, and store the random number sequence on an encrypted electronic crosswalk file. Access to this information will be limited to approved study staff. Participants will be notified to which arm they have been randomized at the completion of the baseline assessment.

Study principal investigators (KH and MAC) will be blinded to participant allocation. Dr. Cucciare will be able to remain blinded despite serving as contact for participants experiencing adverse events. Reasons for this are that Dr. Cucciare has conducted multiple treatment evaluation studies and the rate of contact from participants experiencing adverse events is low or none. In the event that Dr. Cucciare is contacted by a study participant, he will unblind himself, if needed, to the participant's condition to ensure they receive appropriate help or services. However, he will remain blinded to the remaining participants.

Experimental condition

Participants randomized to the experimental condition will receive access to the EMPOWER for Veterans website via telephone, email and/or mail. Study participants will be able to access the EMPOWER for Veterans protocol on their smartphone, desktop, or tablet. The EMPOWER for Veterans protocol will consist of: (a) a self-assessment of risks associated with long-term benzodiazepine use, (b) information on the evidence for benzodiazepine-related harms, (c) knowledge statements designed to create ambivalence about the safety of benzodiazepines, (d) education about drug interactions, (e) vignettes of peers who have successfully stopped using benzodiazepines to support participants' self-efficacy to change, (f) information about equally or more effective therapeutic alternatives for sleep difficulties and/or anxiety, and (g) recommendations for self-tapering (see Appendix A for example of EMPOWER paper version for specific components).

Participants randomized to the EMPOWER condition will be provided information about how to dispose of any unwanted benzodiazepines should they choose to reduce or discontinue their medication. Specifically, we will share with the participant that they may contact their primary care doctor to obtain a Medication Disposal Patient Kit and/or identify nearby locations (via the website artakeback.org) for the Arkansas medication takeback program.

To produce the taper schedule, participants will enter their current benzodiazepine dosage into the EMPOWER website. A tapering schedule will then be generated based on the self-reported number of tablets that the patient is taking each time they take their medication. The tapering schedule will last up to 21 weeks with each day's target pill consumption graphically displayed on a calendar (e.g., full-pill, half-pill, quarter pill, or no pill).

The EMPOWER for Veterans website will also include practical tips for managing the taper process including when to contact one's primary care physician, along with CAVHS and Minneapolis VA pharmacy contact information. Study Participants will be notified of need to obtain a pill cutter along with instructions to contact pharmacy to obtain pill cutter if they do not already have one. The study team will not be providing pill cutters to study participants. We learned from the previous Aim 1 focus groups with Veterans, that most Veterans know how to

obtain a pill cutter on their own since they are easily and cheaply obtainable online or through pharmacies.

Control condition
Participants assigned to the control condition will be asked to continue to follow any provider recommendations regarding benzodiazepine use (i.e., treatment-as-usual). Control participants will also be informed that access to the EMPOWER for Veterans materials will be made available to them at the end of the study (i.e., at 12-months post randomization).

Data Collection
Participants in both conditions will be asked to complete a baseline and 6-month assessment. We also plan to collect data from the CDW on participants' benzodiazepine use at baseline, six-month and 12-month follow-up.

Risks:
The risks associated with study participation include the possibility that a few of the questions (e.g., those inquiring about anxiety symptoms) may cause some psychological discomfort; however, based on our experience in prior VA studies, we expect this to be minimal. It is also possible that Veterans may experience some discomfort with tapering their benzodiazepines. The EMPOWER tool for Veterans will encourage participants to discuss any concerns they might have with discontinuing/reducing their medication with their providers.

To mitigate risks associated with medication tapering a CPRS flag and/or CWAD posting will be created depending on the site.
CAVHS: Study staff will enter a flag in CPRS for Veterans enrolled to indicate study participation along with information that study participants randomized to the EMPOWER intervention arm may choose to self-taper benzodiazepines while enrolled in this study. The flag will also include a link to the EMPOWER site.
Minneapolis VA: Study staff will, in lieu of a flag, will create a post in the Clinical Warning/ Advance Directive (CWAD) Posting. This will serve as a mechanism of notifying healthcare providers that the Veteran is participating in the EMPOWER study.

***Benefits**:*
For Veterans assigned to EMPOWER for Veterans, benefits may include help with discontinuing/reducing their benzodiazepines use and/or giving them information about alternative strategies to help with sleep/anxiety (common indications for prescribing benzodiazepines). Also, Veterans often find it gratifying to participate in efforts that can help other Veterans in need. To that affect, it is believed that this research will help other Veterans reduce or cease the use of benzodiazepines and improve some functional outcomes, like anxiety, sleep quality, and overall health/quality of life.

5.2 Recruitment Methods

See Section 5.1

5.3 Informed Consent Procedures

The informed consent process will be conducted over the telephone by IRB approved research staff who have experience consenting Veterans for intervention studies within VHA. Following the informed consent discussion using the IRB approved informed consent document, Veterans interested in participating in the study will be asked to complete and sign informed consent document and HIPAA authorization forms via the approved methods described below, according to their preference and access to email, printing, and scanning. For CAVHS Veterans

enrolled in the study, the informed consent and HIPAA authorization documents will be scanned into CPRS. A research flag and enrollment note will be entered into CPRS. This note will include identification of the IRB approved mechanism used to obtain documentation of informed consent.

Study staff will obtain documentation of informed consent and HIPAA authorization from individuals who are interested in participating in one of 3 ways: Return-by-Mail, Return-by-Digital Image Capture, Return-by-DocuSign

For Minneapolis study participants, CAVHS research staff will access the study participant's Minneapolis CPRS medical record and create a CWAD posting to include the CAVHS IRB required research flag information. Additionally, study staff will enter a progress note to include templated information required in CAVHS participants' enrollment note adding the study participant's primary care provider (PCP) as an additional signer. This will serve as a mechanism to specifically notify the PCP that the Veteran is participating in the study.

5.3.1 Return by DocuSign. DocuSign is a VA cloud Software as a Service (SaaS) solution approved for use in VA research for the purpose of sending digital documents to patients and collection of their signatures. After completing screening questions, the study team contact will send a DocuSign envelope (email containing links to the study documents) to interested potential participants. The email will contain a reminder for the recipient to not sign the documents prior to the scheduled contact time to review the documents and have questions answered.

5.3.2 Return-by-Mail. Veterans who are interested in participating in the study and who have, or can print, or can receive a faxed copy of the informed consent and HIPAA authorization forms may complete, sign and date these forms and return them to the study team in the provided postage paid envelope. Upon receipt, the study team member who conducted the informed consent process will countersign the ICF and return copies of the ICF and HIPAA authorization to the Veteran via mail. The baseline interview may be initiated after receipt of the signed ICF and HIPAA documents by the study team.

5.3.4 Return-by-Digital Image Capture. Veterans who are interested in participating in the study and who have, or can print, or can receive a faxed copy of the informed consent and HIPAA authorization forms may complete, sign and date these forms and return via:
Email by scanning the digital image or photos of the completed pages of the ICF and HIPAA Authorization forms and sending the images back to the research team via a VA Outlook account, using their personal email account or **Text Message** via cell phone. Veterans using email will be encouraged to use Azure RMS to securely transmit scanned pictures and images back to the study team, however, they cannot be required to use Azure RMS to return documents to the study team.

Veterans who choose to use their personal computers or phones to send images of their consent/ HIPAA documents to the study team via text or unencrypted e-mail, will be told (prior to sending the images) that there is a risk when sending sensitive information in an insecure manner. Study staff will advise participants to retain the signed copies of their ICF and HIPAA Authorization forms for their records and recommend that they delete the emails/texts from their sent items after sending.

Upon receipt, the study team member who conducted the informed consent process will countersign the ICF. The baseline interview may be initiated after receipt of the signed ICF and HIPAA documents by the study team.

5.4 Inclusion/Exclusion Criteria

See Section 5.1

5.5 Study Evaluations

We preregistered two primary outcomes: **cessation of benzodiazepines and dose reduction of benzodiazepines at six-month follow-up.** Cessation will be defined as the absence of a benzodiazepine prescription renewal in the six months prior to follow-up, and dose reduction will be defined as a 25% or greater decrease in benzodiazepine use that has been sustained for three months or more at follow-up. Cessation will be defined as an absence of a benzodiazepine prescription at the time of the 6-month follow-up that was sustained for three consecutive months or more. Dose reduction will be defined as a 25% or greater reduction compared with baseline sustained for three consecutive months or more. All participants who cease benzodiazepines entirely will be included in those who achieve 25% or more reduction.

Assessment of reduction in benzodiazepine use to include differing doses will be converted to lorazepam equivalents and the baseline dose will be defined as the average daily dose in the six-months prior to randomization. These data will be obtained from the CDW. We will also collect participants' self-report data on benzodiazepine use, using a six-month Timeline Followback (TLFB) method (National Institute on Drug Abuse, 2021 [18], to determine the existence of any additional benzodiazepine prescriptions (e.g., from a non-VHA provider) and/or whether any additional benzodiazepine use occurred between baseline and six-month follow-up that was not captured in the administrative data at baseline and six-month follow-up. TLFB is a valid calendar-based method that asks participants to retrospectively recall their drug use (e.g., yes, or no, daily dose) up to two years prior to the interview date (see Appendix B for copy of TLFB).

It is true that CDW prescription medication fill records may not always accurately reflect changes in Veterans' consumption of their medication. Also true is that self-report of medication use is not always accurate. Because all methods for assessing medication use have advantages and disadvantages, we will collect data both from the CDW and the TLFB and we will look at both to determine the outcome.

Pre-registered secondary outcomes are **anxiety symptoms, sleep quality, and overall health and quality of life.** Anxiety symptoms will be measured by the Generalized Anxiety Disorder Scale (Spitzer et al., 2006) [19] (see Appendix C for copy of GAD-7). This seven-item scale asks about the presence and severity of seven symptoms of anxiety (e.g., feeling on edge, having trouble relaxing) and includes an omnibus item rating that is the extent to which any of the seven symptoms has made it difficult "to do your work, take care of things at home, or get along with other people". Items can be summed for an overall rating of anxiety symptoms. Sleep quality will be measured by the Patient-Reported Outcomes Measurement System brief sleep disturbance scale (Yu et al., 2012) [20]. This eight-item scale assesses degree of difficulty falling asleep, degree to which sleep is refreshing, and ability to stay asleep through the night. Items are summed to indicate symptom severity (see Appendix D for copy of sleep assessment).
Overall health and quality of life will be measured by the RAND Veterans Short Form-12 measure (Schalet, 2015) [21], which comprises items assessing global emotional and physical well-being and function (e.g., "How much time in the past four weeks did you have a lot of

energy?", "Compared to one year ago, how would you rate your physical health today?") (see Appendix E for copy of VR-12 form). The final secondary outcome is cessation, or 25% decrease of benzodiazepine use at 12 months; unlike the prior measurements, this will be conducted using databases only.
We will also be assessing participants alcohol use using the three-item Alcohol Use Disorders Identification Test-consumption items, readiness to change medication use using the Readiness Rule, and symptoms of benzodiazepine dependence using the Severity of Dependence Scale. These tools/scales are included in the baseline and 6-month follow-up assessments. Veteran participants will provide self-report of this information for these measures, during their baseline and 6-month interviews.

To determine whether any intervention effects on benzodiazepine use evident at six-months follow-up are maintained, data on benzodiazepine use will again be gathered at 12-month follow-up using VHA centralized databases only (resource constraints prevent assessing this by interview). No other 12-month outcome data will be gathered.

Participant outreach: To conduct 6-month follow up assessments, study staff will attempt to contact Veterans by phone for three months following the date in which their 6-month follow-up was due. We are using this approach because it has worked well in terms of contacting follow-ups in our prior randomized controlled trials. Should the participant state they no longer wish to participate, study staff will document and no further attempts to contact the participant will be made. In our experience, Veterans appreciate attempts to reach them and do not get upset by these multiple attempts.

Should we not be able to reach the person by phone during this 3-month period following the date of the 6-month assessment, we plan to mail the 6-month assessment to the participant. The goal of this mailing is to obtain data from participants who would be otherwise lost to follow up. This is important to do in an intent to treat analysis as we want to make efforts to collect data from all study participants. We have used this approach in previous studies and have been able to obtain data from participants we might have otherwise categorized as lost to follow-up. Should the participant not respond to outreach efforts they will be considered lost to follow up.

EMPOWER website utilization: The EMPOWER website will also include Google Analytics which will anonymously track the number of independent users of the site along with the number of visits. We have also added a question to the 6-month follow-up asking participants if they accessed the EMPOWER website in the prior 6-months.

5.6 Data Analysis

The randomization scheme will be checked by comparing key baseline measures between the experimental and the control group. If significant differences between groups are identified, we will adjust for these variables in all the models. Baseline variables that are strongly associated with the outcomes will also be included in the corresponding outcome models. Linearity between continuous variables and outcomes will be checked. If the relationship is not linear, other appropriate functional forms (e.g., quadratic) will be used or the variable will be changed to categorical. Proportions of missing values will be assessed, and a multiple imputation method will be used to impute the missing values if necessary and if the missing at random assumption is deemed reasonable.

Descriptive statistics will be calculated to examine data distributions. Tests for distributional assumptions (e.g., normality) for continuous variables will also be performed. Bivariate analyses will be performed for categorical variables using a Chi-square test, for continuous normally

distributed variables using independent t-test and for non-normally distributed continuous variables using nonparametric Wilcoxon rank-sum test.

For multivariate analysis for the main outcomes of benzodiazepine discontinuation and 25% dose reduction at follow-ups, we will use generalized estimating equations (GEE) models or generalized linear mixed models to examine the effect of EMPOWER for Veterans on the proportions of participants with discontinuation and dose reduction with logit link and compound symmetry correlation structure (in GEE) due to repeated measures within each participant. All the models will include the indicator variable for the EMPOWER for Veterans versus control conditions, time representing six- and 12- month follow-ups and covariates identified in bivariate analysis. The baseline benzodiazepine use (i.e., average daily dose) variable will also be included. The interaction between the indicator variable and time will be checked and dropped if non-significant at the significance level of 0.05.

The same method will be used to examine any impact of the intervention on the three secondary outcomes at baseline and six-month follow-up (anxiety, sleep quality, and quality of life) with identity link and the same correlation structure if the normality assumption is met. Should the normality assumption be violated, other distributions and link functions will be specified. The models will include the same indicator variable for EMPOWER for Veterans versus the control condition, time for baseline and 6-month follow-up and covariates identified in bivariate analysis. Same interaction term will be checked and dropped if non-significant. SAS 9.4 will be used in all the analyses.

5.7 Withdrawal of Subjects

We do not anticipate withdrawing participants without their consent for this aim. However, should a participant voluntarily withdraw from the study, we will seek their permissionto use their data in the analysis. Should they not agree, we will remove their data from the analysis and store their data with study records. We do not plan to follow-up with participants who are withdrawn by study staff or voluntarily withdraw from the study. Per VA, participation in research is voluntary and study participants will not lose any benefits to which they are entitled and will continue to receive care. Should a Veteran be withdrawn from the study, we will be clear in communicating with the Veteran that they are still eligible for VA care.

6.0 Reporting

Should an adverse event occur, study staff will complete a study form that will include the participant’s study number and all relevant information about the adverse event. All staff will be required to immediately notify Dr. Cucciare about the occurrence of a serious or significant adverse event. Upon notification of the adverse event, Dr. Cucciare will take the appropriate action. A study nurse will be available to assess and take appropriate action. For example, if the adverse event is a medical issue (e.g., participant concern about symptoms of medication withdrawal), Dr. Cucciare or the study nurse will immediately notify the staff physician, Dr. Richard Owen about the concern. Dr. Owen will contact the participant to address any concern and provide medical advice and recommendations, if needed. Following this contact, Dr. Cucciare, a study nurse or Dr. Owen will follow-up with the participant within 24 hours of the initial contact. This protocol will apply for both CAVHS and Minneapolis VAMC sites.

Dr. Cucciare will also be responsible for reporting any protocol deviations to IRB within 24 hours of their occurrence Case Report forms will be completed immediately by the study personnel responding to the event and submitted to the IRB within 24 hours of the occurrence.

An annual report will be submitted to the local Institutional Review Board and project officer summarizing all adverse events. These procedures for reporting adverse events will apply to adverse events occurring during the baseline, follow-up phases of the clinical trial and during the conduct of the process evaluation.

7.0 Privacy and Confidentiality

This study will access PHI for recruitment under a waiver of HIPAA authorization. This study will collect PHI from study participants with their authorization. PHI will include names and social security numbers. All PHI collected will be stored in locked cabinets under triple lock (cabinet, office, front door). Study staff collecting and storing PHI will be trained in steps to maintain participants' data security (e.g., using secure methods for storage such data). Any electronic PHI (e.g., birthdates) will be stored on an encrypted, password protected server behind the VA firewall. PHI will be coded and maintained in data crosswalk that will also be stored on an encrypted, password protected server. Research Investigator files will be destroyed six years after the end of the fiscal year when the research project has been completed per Records Schedule DAA-0015-2015-004. The ISSO/PO and ACOS/RD will be notified immediately if there is inadvertent loss or disclosure of research data.

8.0 Communication Plan

This protocol and all related documents shall be approved by the IRB and R&D Committee prior to enrollment of any subjects. The informed consent process shall be conducted only by appropriately qualified study personnel. The privacy and confidentiality of subjects shall be respected by all study personnel. The research team will regularly meet to discuss the study's progress and address any adverse events or unanticipated issues. The study coordinators will ensure all applicable IRB and RDC approvals are in place and will be in continuous communication with the principal investigators, Drs. Michael Cucciare and Keith Humphreys.

9.0 References

1. Bachhuber, M.A., Hennessy, S., Cunningham, C.O., Starrels, J.L. Increasing benzodiazepine prescriptions and overdose mortality in the United States, 1996-2013. *Am J Public Health*. 2016; 106: 686-688.
2. Lembke, A., Papac, J., Humphreys, K. Our other prescription drug problem. *New Engl J Med*. 2018; 378(8): 693-695.
3. Park, T.W., Larochelle, M.R., Saitz, R., Wang, N., Bernson, D., Walley A.Y. Associations between prescribed benzodiazepines, overdose death and buprenorphine among people receiving buprenorphine. *Addiction*. 2020; 115(5): 924-932.
4. Sun, E.C., Dixit, A., Humphreys, K., Darnell, B.D., Baker, L.C., Mackey, S. Association between concurrent use of prescription opioids and benzodiazepine with overdose: A retrospective analysis. *BMJ*. 2017; 356: j760.
5. U.S. Department of Veterans Affairs. Veterans Health Administration. PBM Academic Detailing Service. Benzodiazepine risks: Are you aware of the possible risks from taking benzodiazepines? https://www.pbm.va.gov/PBM/AcademicDetailingService/Documents/Benzodiazepine_Provider_AD_%20Risk_Discussion_Guide.pdf. Accessed January 13, 2021
6. Lader, M. Benzodiazepines revisited--will we ever learn? *Addiction*. 2011; 106(12): 2086-2109.

7. Gould, R.L., Coulson, M.C., Patel, N., Highton-Williamson, E., Howard, R.J. Interventions for reducing benzodiazepine use in older people: Meta-analysis of randomized controlled trials. *Br J Psychiatry*. 2014; 204(2): 98-107.
8. Garnett, C., Oldham, M., Angus, C., Beard, E., Burton, R., Field, M., et al. Evaluating the effectiveness of the smartphone app, Drink Less, compared with the NHS alcohol advice webpage, for the reduction of alcohol consumption among hazardous and harmful adult drinkers in the UK at 6-month follow-up: Protocol for a randomised controlled trial. *Addiction*. 2020 Oct 17. doi: 10.1111/add.15287. Epub ahead of print.
9. Cheung, Y.T.D., Chan, C.H.H., Ho, K.S., Fok, W.P., Conway, M., Wong, C.K.H., et al. Effectiveness of WhatsApp online group discussion for smoking relapse prevention: Protocol for a pragmatic randomized controlled trial. *Addiction*. 2020; 115(9): 1777-1785.
10. Cunningham, J., Wild, T.C., Cordingley, J., van Mierlo, T., Humphreys, K. A randomized controlled trial of Internet-based intervention for alcohol abusers. *Addiction*. 2009; 104(12): 2023-2032.
11. Cunningham, J.A., Neighbors, C., Wild, T.C., Humphreys, K. Ultra-brief intervention for problem drinkers: Results from a randomized controlled trial. *PLoS One*. 2012; 7(10): e48003.
12. Erbes, C.R., Stinson, R., Kuhn, E., Polusny, M., Urban, J., Hoffman, J., et al. Access, utilization and interest in mHealth applications among veterans receiving outpatient care for PTSD. *Mil Med*. 2014; 179(11): 1218-1222.
13. Klee, A., Stacy, M., Rosenheck, R., Harkness, L., Tsai, J. Interest in technology-based therapies hampered by access: A survey of veterans with serious mental illnesses. ***Psychiatr Rehabil J. 2016; 39***(2): 173-179.
14. Damschroder, L.J, Hagedorn, H.J. A guiding framework and approach for implementation research in substance use disorders treatment. *Psychol Addict Behav*. 2011; 25(2); 194-205.
15. Tannenbaum, C., Martin, P., Tamblyn, R., Benedetti, A., Ahmed, S. Reduction of inappropriate benzodiazepine prescriptions among older adults through direct patient education: The EMPOWER cluster randomized trial. *JAMA Intern Med*. 2014; 174(6); 890-898.
16. Correspondence with VA Pharmacy Benefits Management, 2017
17. Boumparis, N., Karyotaki, E., Schaub, M.P., Cuijpers, P., Riper, H. Internet interventions for adult illicit substance users: A meta-analysis. *Addiction*. 2017; 112(9): 1521-1532.
18. National Institute on Drug Abuse. CTN Common Data Elements. Instrument: Timeline Followback method assessment. https://cde.drugabuse.gov/instrument/d89c8e23-16e5-625a-e040-bb89ad43465d. Accessed January 13, 2021
19. Spitzer, R.L., Kroenke, K., Williams, J.B.W., Lowe, B. A brief measure for assessing generalized anxiety disorder: The GAD-7. *Arch Intern Med*. 2006; 166(10): 1092-1097.
20. Yu, L., Buysse, D.J., Germain, A., Moul, D.E., Stover, A., Dodds, N.E., et al. Development of short forms from the PROMIS Sleep Disturbance and Sleep Related Impairment item banks. *Behav Sleep Med*. 2012; 10(1), 6-24.
21. Schalet, B.D., Rothrock, N.E., Hays, R.D., Kazis, L.E., Cook, K.F., Rutsohn, J.P., et al. Linking physical and mental health summary scores from the Veterans RAND 12-Item Health Survey (VR-12) to the PROMIS Global Health Scale. *J Gen Intern Med*. 2015; 30(10): 1524-1530.
