# Supplementary material for: Electronic Intervention for Patient-Managed Benzodiazepine Tapering: A Randomized Clinical Trial
Source: JAMA Netw Open. 2026 Jan 14;9(1):e2551807. doi: 10.1001/jamanetworkopen.2025.51807 (PMC12805446; doi:10.1001/jamanetworkopen.2025.51807)
Supplement: Supplement 2. — Data Sharing Statement [file jamanetwopen-e2551807-s002.pdf]

Data Sharing Statement

Humphreys. Electronic Intervention for Patient-Managed Benzodiazepine Tapering. *JAMA Netw Open*. Published January 14, 2026. doi:10.1001/jamanetworkopen.2025.51807

Data
Additional Information: Registered at ClinicalTrials.Gov (NCT04572750) on October 1, 2020.
Data available: Yes
Data types: Data dictionary, Deidentified participant data
How to access data: knh@stanford.edu
When available: With publication

Supporting Documents
Document types: None

Additional Information
Who can access the data: knh@stanford.edu
Types of analyses: Any scholarly purpose
Mechanisms of data availability: With suipport
Any additional restrictions: N/A
